# Supplementary material for: Dramatic resurgence of malaria after 7 years of intensive vector control interventions in Eastern Uganda
Source: PLOS Glob Public Health. 2024 Aug 29;4(8):e0003254. doi: 10.1371/journal.pgph.0003254 (PMC11361418; doi:10.1371/journal.pgph.0003254)
Supplement: Attachment — Submitted filename: Responses to reviewers comments-1.docx [file pgph.0003254.s001.docx]

Cover letter

We appreciate your careful review of our manuscript titled “**Dramatic resurgence of malaria after 7 years of intensive vector control interventions in Eastern Uganda**”. Below is a list of comments from the reviewers followed by our responses with reference to changes made to the manuscript.

Journal Requirements:

1. Please provide separate figure files in .tif or .eps format only and remove any figures embedded in your manuscript file. Please also ensure all files are under our size limit of 10MB.

**Response:** This has been done.

Reviewer #1:

1. It would of value to add some discussions around any potential variations (or not) in rainfall and temperatures that could also have an influence in entomological variables. Similarly, I don't think I saw any information about the transmissions seasons in the study areas. It's possible that there is no clear high transmission period(s) in the neither districts - but still good information for context.

**Response:** We did not collect data on rainfall or temperature in this study but have added the following additional details about the transmission seasons in the study areas:

*“This area is characterized by perennial transmission with two seasonal peaks following the rainy seasons.”*

2. Consider rephrasing lines 340-344, I think the "each of the first 7 months of observation" should be replaced with simply "the first 7 months of observations".

**Response:** This change has been made.

3. In a few places, you introduce description with "briefly" - I don't think it necessary.

**Response:** We have removed “briefly” where appropriate throughout the manuscript.

Reviewer #2: 

1. Line # 28-29: Clearly from your results, IRS with carbamates & Actellic was implemented during the 5years of dramatic decrease. As such, the use of the term “following 5 years of…” may be misleading as it suggests that IRS was implemented for 5years after which, there was a dramatic decrease. Please consider using a more appropriate term such “during 5 years …” to avoid confusion.

**Response:** This change has been made

2. Line # 45: It may not be clear to the reader of the abstract when you report average monthly cases without a specific period covering the months for which the average was generated.

**Response:** We recognize that the range of dates to which these averages are referring to is important to include in the abstract. However, we were unable to edit the abstract due to the strict word limit.

3. Line # 57: You summarize sporozoite rate simply as approximately 50%, again without indicating the period being considered, given that you define distinct durations for consideration in the paper. One would have expected sporozoite results to be presented by the durations under consideration.

**Response:** These details have been added.

4. There are many punctuation issues in the paper that may need to be cleaned up. For instance,

Line # 70: Between 2000 and 2015, it was estimated that …

Line # 80: … has been slow and difficult to sustain, …
Line# 129: As in Tororo prior to 2013, vector …
Line # 187-189: … clinic visit, …
And many, many other places that punctuation needs to be improved.

**Response:** These punctuation issues have been corrected.

5. Importantly also, your punctuation around in-text citations needs consideration. It’s rather strange that your punctuation comes before your in-text citations. Please double check PGPH recommended on citation. This also goes for the use of “(“ rather than “[“ as recommended by the PGPH journal.

**Response:** The punctuation around in-text citations has been corrected.

6. There is not a clear section in the paper, either in the methods, results or discussions that addresses the part of the study aim pertaining to “causes of resurgence”. This may need to be clarified.

**Response:** At the time we designed this observational cohort study we were not aware that a resurgence of malaria would occur in Tororo District. As explained in the methods section, after our initial enrollment period it became clear that the burden of malaria on the Tororo side of the border was higher than expected and therefore we decided to enroll additional houses in Tororo farther away for the border. Once it became clear that Tororo was experiencing a marked resurgence in the burden of malaria, we attempted to best utilize the data we were collecting to explain the potential causes of the resurgence. Indeed, we devote much of the discussion section addressing what we thought were the most likely causes of the resurgence.

7. Line # 90-91: This sentence may need to be simplified to avoid confusing the reader on what happened when. For instance, stating this as “Comparing key malaria indicators measure prior to IRS (2011-14 and after two rounds of LLIN as well as 5 years of sustained IRS (2017-19), …” or simpler, would help the reader know when there was or the wasn’t IRS and/or LLIN.

**Response:** This recommended change has been made.

8. Line # 94: The reported “marked shift in predominant vector” may need a reference. It’s also not clear whether this marked shift was all through 2011-19 or that you’re referring to 2017-19.

**Response:** A reference has been added to this statement and the wording has been edited to provide clarity as to the time periods being compared.

9. Line #118-134: Lots of information is provided here without a single reference. Certainly, one would not expect that all these LLIN and IRS activities were part of your study or its data collection in the field. Please provide appropriate sources of this valuable information.

**Response:** A reference has been added describing prior vector control interventions implemented in the study area.

10. Line # 130: The sentence may need to be corrected. “Universal distribution campaigns were … “

**Response:** This has been edited.

11. Line #155-158: It would be helpful to provide a brief rationale for the seemingly dissimilar definition of near-border households between the two districts. In one, it was 1.9 – 3.2Km and in the other 0.7-3.5Km areas

**Response:** We were unable to enroll houses any closer to the border because of a river/swamp area along the border that is uninhabitable. This detail has been added.

12. It’s also not quite clear why in Busia, there was no category of households characterized as “away from the border”. Was the general assumption that every where outside of IRS cover would be the same? This would be strange, given that you indicated that your plan was to examine the gradient over this transmission area. If not, this may be a limitation to your capacity to fully understand the gradient of interest.

**Response:** As described in the methods section, we initially only enrolled houses on either side of the border between the two districts with the goal of comparing the epidemiology of malaria between these two areas (with and without IRS). However, preliminary data indicated that malaria burden was surprisingly similar between these two areas. There to capture more of a “gradient in transmission intensity”, we enrolled additional houses but only had the resources to do this farther away from the border on the Tororo side, which we felt would be of greater interest.

13. Line # 166: From an ethical stand point, were the 4 households that were replaced included in any of the analyses, be it even for the duration prior to their withdrawal? This may need to be made clear because, they are not expected to be included at all, given their withdrawal of consent.

**Response:** 4 households were withdrawn from the study after all individual participants from these households were withdrawn from the study (e.g. everyone in the household had moved out of the study area). This has been clarified. In no cases were participants prematurely withdrawn from the study and a request was made not to use their data.

14. Line #281: As seen from your Figure 3 A, episodes / person years were already on an upward trend. Given this, how did you define “increase accelerated” or are you referring to the increase not having been slowed or disrupted? This terminology may be misleading.

**Response:** This section refers to Figure 2, not Figure 3A. We maintain that our description of the temporal changes presented in Figure 2 is appropriate.

15. Line # 284-285: As your Figure 3A clearly shows, malaria decline doesn’t appear to be tied to actellic alone. From your results, even the location with no IRS showed the same level of decline of malaria burden. You may want to tone this claim down to be more consistent with your results.

**Response:** Again, this section refers to Figure 2, not Figure 3A.

16. Line #315: It’s not clear or indeed confusing why after indicating that the duration of assessment of incidence as between Apr-2021 – Sep-2023 in the text before this sentence, you then start describing this part from Feb-Aug 2021, which included a large chunk of a duration outside of the stated study duration.

**Response:** Feb-Aug 2021 was chosen to represent a “baseline” period away from the border in Tororo when the incidence of malaria was relatively low and had not yet started to increase. Separately, we chose to compare 6-month time periods from April 2021 through September 2023 so that we could make direct, quantitative comparisons over time between the 3 geographic regions. We chose to divide calendar time in this way to provide 5 complete 6 month time periods from all 3 geographic areas through the end of the study period.

17. Line #387: Please clarify why since you set out to evaluate causes of resurgence including entomological measures as indicated in your methods, that then you lacked the precision to evaluate temporal trends in proportions of mosquitoes infected with sporozoites.

**Response:** These details have been added.

18. Line #517-518: It’s also surprising that your study {that was “designed to characterize the resurgence and explore potential causes…”, as stated in prior to this, did all the entomological procedures extensively explained and later elaborated on in the discussion} reports the limitation of susceptibility that did not utilize mosquitoes in the study districts. You may consider revisiting the strong worded aim of the study to this effect. Otherwise, it appears that this study was designed to assess something else and possibly not exactly the resurgence. If not, then this limitation is not expected.

**Response:** We simply did not have the resources to conduct susceptibility testing from mosquitos collected from our study districts and had to use mosquitoes collected from a neighboring study districts that had been collected as part of a separate study. This detail has been added.

19. Line # 524: As it is, the conclusion drawn around resurgence being geographically corelated with change in IRS formulation is not supported by your results. For instance, 1) Your results indicated a fairly similar temporal trend in burden (episodes per 1000-person years) across site-geographies. 2) You didn’t have similar strata in Busia as you did in Tororo (near versus away from border) to adequately examine geographical variations.

**Response:** We disagree with this statement. Our data clearly shows a marked resurgence in Tororo district following the change in IRS formulation from Actellic to Fludora Fusion (both using health facility based data and data from our cohort study). In contrast, there was no resurgence of malaria accrss the border in Busia District where IRS has never been implemented.

20. Line # 525: Stating that resurgence was “correlated” with change in IRS may also be an overstatement as clearly shown in your results. Importantly, FF IRS occurred in the middle of a heavily developing burden peak. It’s would not be true that the peak was accelerated following this IRS change.
One would argue, rather strongly and also consistent to what you stated elsewhere in the paper and supported by your Figure 3A, that there is an apparent slower action of FF, which delays and/or dampens the slope after the peak occurring during the FF spray round.

**Response:** Please see the response to comment # 19 above. We agree that the dynamics of different rounds of IRS and malaria burden are complicated. We simply make the conclusion that there was a strong temporally and geographic correlation between changes in IRS formulations and the observed malaria resurgence and offer some hypotheses on a potential causal link between these. We took care to not imply that we had proven a causal association.

21. Line # 533-534: Given your indication that your program has been conducting surveillance in this same site since 2011. Are you, by recommending that surveillance data should be timely…” implying that your surveillance data was not timely or available to the program to foster a well-coordinated implementation of this round of IRS. Rather than this rather distal conclusion, one would have expected some aspects of the conclusion to address the part of the aims of your study concerning the causes of this resurgence, which is currently missing.

**Response:** We did not design this study to identify the causes of the resurgence. Rather the fortunate existence of our surveillance work in this area provided a unique opportunity to characterize the resurgence (which was unexpected). In the conclusion section we simply highlight the value of comprehensive malaria surveillance (which is often lacking) and the role it can play in policy making.

22. Figure 1: The legend refers to PRISM1 and 2 study sites yet there seems to be only one site (purple) on the map.

**Response:** This figure is correct. The PRISM 1 and 2 studies were conducted at the same site.

23. Figure 4: Your use of the same exact colors that represent sites in other Figures for vector types may need to be revisited to enable readers better distinguish these results. Please consider using the same y-axis scale in this Figure, especially for A and B, as they are both referring to Tororo.

**Response:** We have changed the color scheme of this figure so that it is distinct from the other figures. We prefer not to change the scale of the y-axis, otherwise it is difficult to appreciate the relative changes in vector densities. However, we have added to the figure legend to make it clear that the scale of the y-axis differs by study site.

24. Figure 5: The x-axis label of “Days post exposure” seems inappropriate unless you intend to represent 1hr in terms of days. It could be better stated as “time post exposure”.

**Response:** This has been changed to “Time post exposure”

25. Author contributions: It appears that there are so many co-authors without specified contributions to the paper. Say for instance, Jimmy Opigo, Teun Bousema, Phil Rosenthal, … and many others.

**Response:** All of the co-authors listed played a key role in reviewing and editing the manuscript as well as other aspects that were not provided as categories under authors’ contributions.

26. Table 3: There are several N quantities presented that are not fully explained. For instance, there’s N* in the column heading, then *N in the footnote, and then N when describing VD. These may need to be clarified clearly spelling out which is which.

**Response:** The quantities presented in Table 3 have been clarified.

27. In a number of your tables, why is there inconsistent use of 2 and in other cases 1 decimal places, especially Tables 2 and 3? Please consider making this consistent.

**Response:** The number of decimal places chosen based on the metric and what we felt was the necessary level of precision.
