# Supplementary material for: Dramatic resurgence of malaria after 7 years of intensive vector control interventions in Eastern Uganda
Source: PLOS Glob Public Health. 2024 Aug 29;4(8):e0003254. doi: 10.1371/journal.pgph.0003254 (PMC11361418; doi:10.1371/journal.pgph.0003254)
Supplement: Attachment — Submitted filename: Responses to reviewers comments round 2_May 17th_2024.docx [file pgph.0003254.s002.docx]

May 19^th^, 2024

Response to reviewers- Rebuttal letter

We appreciate your careful review of our manuscript titled “**Dramatic resurgence of malaria after 7 years of intensive vector control interventions in Eastern Uganda**”. Below is a list of comments from the editor and reviewers followed by our responses with reference to changes made to the manuscript.

Journal Requirements:

1) Please review your reference list to ensure that it is complete and correct. If you have cited papers that have been retracted, please include the rationale for doing so in the manuscript text, or remove these references and replace them with relevant current references. Any changes to the reference list should be mentioned in the rebuttal letter that accompanies your revised manuscript. If you need to cite a retracted article, indicate the article’s retracted status in the References list and also include a citation and full reference for the retraction notice.

**Response:** Our reference list has been reviewed and is complete and correct.

2) Please ensure that Funding Information and Financial Disclosure Statement are matched.

3) In the Funding Information you indicated that no funding was received. Please revise the Funding Information field to reflect funding received.

**Response:** We have double checked the Financial Disclosure Statement and found that it is consistent with Funding Information provided in the manuscript i.e. *This project was funded by the US National Institutes of Health as part of the International Centers of Excellence in Malaria Research (ICEMR) program (U19AI089674).*

4) Some material included in your submission may be copyrighted. According to PLOS’s copyright policy, authors who use figures or other material (e.g., graphics, clipart, maps) from another author or copyright holder must demonstrate or obtain permission to publish this material under the Creative Commons Attribution 4.0 International (CC BY 4.0) License used by PLOS journals. Please closely review the details of PLOS’s copyright requirements here: PLOS Licenses and Copyright. If you need to request permissions from a copyright holder, you may use PLOS's Copyright Content Permission form.

Please respond directly to this email or email the journal office and provide any known details concerning your material's license terms and permissions required for reuse, even if you have not yet obtained copyright permissions or are unsure of your material's copyright compatibility.

Potential Copyright Issues:

Fig 1: please (a) provide a direct link to the base layer of the map (i.e., the country or region border shape) and ensure this is also included in the figure legend; and (b) provide a link to the terms of use / license information for the base layer image or shapefile. We cannot publish proprietary or copyrighted maps (e.g. Google Maps, Mapquest) and the terms of use for your map base layer must be compatible with our CC-BY 4.0 license.

Note: if you created the map in a software program like R or ArcGIS, please locate and indicate the source of the basemap shapefile onto which data has been plotted.

If your map was obtained from a copyrighted source please amend the figure so that the base map used is from an openly available source. Alternatively, please provide explicit written permission from the copyright holder granting you the right to publish the material under our CC-BY 4.0 license.

Please note that the following CC BY licenses are compatible with PLOS license: CC BY 4.0, CC BY 2.0 and CC BY 3.0, meanwhile such licenses as CC BY-ND 3.0 and others are not compatible due to additional restrictions.

If you are unsure whether you can use a map or not, please do reach out and we will be able to help you. The following websites are good examples of where you can source open access or public domain maps: 
* U.S. Geological Survey (USGS) - All maps are in the public domain. ([http://www.usgs.gov](https://urldefense.com/v3/__http:/www.usgs.gov__;!!LQC6Cpwp!pVOc90K5bWeeFOCKkL2oKLLBWxEn1ZVYmsYhnytyAQswZzLTHZul3QpTga-vEpKXc--g4oGmYTPZZ9oeNsuU_aQ$)) 
* PlaniGlobe - All maps are published under a Creative Commons license so please cite “PlaniGlobe, [http://www.planiglobe.com](https://urldefense.com/v3/__http:/www.planiglobe.com__;!!LQC6Cpwp!pVOc90K5bWeeFOCKkL2oKLLBWxEn1ZVYmsYhnytyAQswZzLTHZul3QpTga-vEpKXc--g4oGmYTPZZ9oejeArjsE$), CC BY 2.0” in the image credit after the caption. ([http://www.planiglobe.com/?lang=enl](https://urldefense.com/v3/__http:/www.planiglobe.com/?lang=enl__;!!LQC6Cpwp!pVOc90K5bWeeFOCKkL2oKLLBWxEn1ZVYmsYhnytyAQswZzLTHZul3QpTga-vEpKXc--g4oGmYTPZZ9oeA9DJmMI$)) 
* Natural Earth - All maps are public domain. ([http://www.naturalearthdata.com/about/terms-of-use/](https://urldefense.com/v3/__http:/www.naturalearthdata.com/about/terms-of-use/__;!!LQC6Cpwp!pVOc90K5bWeeFOCKkL2oKLLBWxEn1ZVYmsYhnytyAQswZzLTHZul3QpTga-vEpKXc--g4oGmYTPZZ9oeaq0Yd2w$))

**Response:** The map in Figure 1 was created using shapefiles of the districts, parishes, and villages of Uganda obtained from the Humanitarian Data Exchange v1.80.0 PY3, linked here: [https://data.humdata.org/dataset/uganda-administrative-boundaries-as-of-17-08-2018.](https://data.humdata.org/dataset/uganda-administrative-boundaries-as-of-17-08-2018) Explicit data licensing terms can be found here: [https://data.humdata.org/faqs/licenses.](https://data.humdata.org/faqs/licenses) This information has been added to the Figure 1 legend.

Additional Editor Comments:

Thank you for this revision. While almost all peer reviewer feedback has been addressed, I would like to request further clarifications on the following:

1) Question 16 from reviewer #2 was not fully addressed, and I agree that it is a little confusing to mention that incidence was analysed in 6-month chunks, then in the next sentence to describe incidence in a way that does not use the same 6-month breakdown. Perhaps a clarification is needed at the start of the sentence to emphasize that you are now assessing the monthly incidence estimates.

**Response:** We apologize for this confusion. We have rearranged the wording of this paragraph such that the description of how we analyzed the data in 6-month intervals was moved to just before we begin summarizing the results based on these 6-month intervals.

2) I suggest to adjust the colour schemes for IRS types used in figure 2 to match those in figures 3 & 4. In addition, is there a particular reason why in figure 2 the whole period is shaded to match specific IRS spray schemes, while in figure 3 & 4 only the period when IRS campaign occurred is shaded? If the intention is to indicate the period of expected residual effectiveness in figure 2, please clarify this in the figure description.

**Response:** The color scheme for Figure 2 has been changed to match Figures 3 & 4. For Figure 2 we chose to shade the whole period (including expected residual effectiveness) to match specific IRS spray schemes as we thought the figure would look “too busy” to include all 11 rounds of the different IRS formulations used from 2014-2023 (which are described in detail in the methods section). In addition, we felt that shading the whole periods provided a better visualization of transition of one IRS formulation to the next. We have edited the legend for Figure 2 to explain the color scheme used for the different IRS formulations.

3) Regarding the response to question 20 from reviewer 2, you stated that you took care not to imply that you had proven a causal hypothesis, however in line 428 you state that the evidence presented does support a causal link. If you do wish to indicate that these evidence are indicative of a causal link between changing IRS chemicals and resurgence, I recommend referring to or framing this around the Bradford-Hill criteria or other causal inference literature using observational study designs.

**Response:** Thank you for this suggesting. We have edited this statement in the Discussion section to include Bradford Hill criteria for deducing causality, recognizing the limitations of our observational study design.
